# Poor immunity status against poliomyelitis in medical students: a semi-anonymous study

**DOI:** 10.1007/s00430-012-0237-2

**Published:** 2012-06-13

**Authors:** Manuel Külshammer, Ute Winke, Monika Frank, Ursula Skali-Lami, Henrike Steudel, Gert Schilling, Jan Felix Drexler, Anna Maria Eis-Hübinger, Bertfried Matz

**Affiliations:** 1 Institute of Virology, University of Bonn Medical Centre, Sigmund-Freud-Str. 25, 53105 Bonn, Germany; 2 Unit of Occupational Medicine, University of Bonn Medical Centre, Sigmund-Freud-Str. 25, 53105 Bonn, Germany

**Keywords:** Polioviruses, Immunity status, Medical students

## Abstract

In spite of almost complete eradication, poliomyelitis continues to be a global threat even in non-endemic countries due to the ever-increasing international travel activities. Health care workers are at a special risk in acquiring pathogens from travelers returning from endemic countries. Polio vaccines are fairly well accepted throughout the German population. Yet, laboratory controls for successful immunization are carried out only sporadically in the general population, and not even the medical staff are routinely tested for polio immunity. The present study was initiated in order to assess the immunity status of young people at the very beginning of their career in clinical medicine. Within their first clinical semester, all students are supposed to undergo an obligatory health check in our Occupational Medicine Unit. A blood sample is taken and sent under a personal code to our diagnostic laboratories for virus serology, and for cryoconservation of residual serum, if available. Within the periods 2004–2006 and 2008–2010, we analyzed sera from 424 and 427 individuals, respectively, for anti-polio types 1, 2, 3 antibodies by a microneutralization assay. In the latest study period, there was a slight increase in the rate of fully protected persons: 63.9 % triple-seropositivity versus 57.1 % in the period 2004–2006. By the end of the second clinical semester, students with low or negative antibody levels (1:<10) were informed, and a (booster) vaccination was recommended.

## Introduction

The progress in eradicating poliomyelitis by vaccination programs has been accompanied by a series of cumbersome setbacks. Wild-type polioviruses types 1 and 3 (WPV) are repeatedly imported from endemic regions into polio-free countries [1]. A large outbreak in Tajikistan in 2010 [2] as well as a recent cluster in China in 2011 [3] demonstrates that poliomyelitis still is more than a merely theoretical problem for global health. A further problem is the emergence of mutant poliovirus strains [4, 5] originating from the live, attenuated oral poliovirus vaccine (OPV), termed vaccine-derived polioviruses (VDPV). For these reasons, health authorities are anxious to keep population immunity at high levels.

As for Germany, a large survey conducted in 1997/1998 covering all age classes and all federal states [6] had revealed a sufficiently high rate of seropositivity against all three types of polioviruses (85 %). It was, therefore, somewhat disappointing when 7 years later (2005) within a cohort of medical students at the University of Frankfurt/Main, only 68 % were found to be completely protected against poliomyelitis [7].

Having concurrently observed a low immunity rate among our patients at the Bonn University Clinics, we started an antibody screening with our medical students. Our first results in 2003 were alarming so that we decided to continue the project with a systematic evaluation of the results from 2004 onwards. The study was designed in a way that allowed the individual students to easily get information about a potential lack of immunity.

## Subjects

A semi-anonymous serological survey among medical students was conducted in the following way: Prior to their training in clinical medicine, all students must undergo a medical examination at our Unit for Occupational Medicine. A blood sample was taken and sent under a personal code to our diagnostic laboratories for mandatory HBV and HCV testing and HIV serology on a voluntary basis. Residual serum samples were stored frozen. For our study, a list with personal codes of students in their second clinical semester was transmitted to the laboratory. Whenever available, the corresponding frozen sera were thawed and analyzed for poliovirus antibodies. In total, 424 sera available in the first period (2004–2006) and 427 sera from the second period (2008–2010) could be analyzed in our study. A list with the codes of all those individuals with a low antibody titer (1:<10) was displayed on our notice board.

## Methods

Antibodies against poliovirus types 1, 2, and 3 were analyzed by a manual microneutralization assay essentially as described earlier for an automated assay [8, 9], using African green monkey kidney cell line VERO in 96-well microtiter plates (50,000 cells per well) and Sabin vaccine virus strains at a challenge dose of 100 TCID50 per well. After 3 days of incubation at 37 °C in a 6 % CO_2_ atmosphere, cytopathic effects were monitored by inverse light microscopy. A titer below 1:10 was considered as potentially non-protective.

## Results

In the first study period (2004–2006), sera from 424 medical students were tested for neutralizing antibodies against the three poliovirus types. Anti-poliovirus type 1 antibodies were found at titers of 1:10 or higher in 355 samples (83.7 %), whereas 69 sera (16.3 %) failed to neutralize at the 1:10 dilution. With poliovirus type 2, the situation was quite similar: 384 sera (90.6 %) were reactive at 1:10 or higher dilutions, and 40 samples (9.4 %) were negative at that dilution. Immunity against type 3 was present (1:10 or above) only in 265 subjects (62.5 %), and low or absent (1:<10) in as many as 159 students (37.5 %).

Evaluation of the data from the second study period (2008–2010) yielded a slight improvement with regard to the poliovirus type 3 immune status, while the type 1 and type 2 immunity rates remained largely unchanged. Out of 427 sera, 362 (84.8 %), 380 (89.0 %), and 290 (67.9 %) had titers (1:10 or more) against poliovirus type 1, type 2, and type 3, respectively. At the 1:10 dilution, 65 sera (15.2 %) failed to neutralize type 1, 47 (11.0 %) failed to neutralize type 2, and 137 (32.1 %) were negative with type 3 (Table 1).

**Table 1:** Poliovirus immunity status of medical students

	Study period	
	2004–2006	2008–2010
Number of subjects	424	427
*Unprotected against*		
Serotype 1	69 (16.3 %)	65 (15.2 %)
Serotype 2	40 (9.4 %)	47 (11.0 %)
Serotype 3	159 (37.5 %)	137 (32.1 %)
Serotype 1 only	18 (4.2 %)	12 (2.8 %)
Serotype 2 only	2 (0.5 %)	3 (0.7 %)
Serotype 3 only	97 (22.9 %)	71 (16.6 %)
Serotypes 1 + 2	3 (0.7 %)	2 (0.5 %)
Serotypes 1 + 3	27 (6.4 %)	24 (5.6 %)
Serotypes 2 + 3	14 (3.3 %)	15 (3.5 %)
Serotypes 1 + 2 + 3	21 (5.0 %)	27 (6.3 %)
Unprotected against one, two, or all serotypes	182 (42.9 %)	154 (36.1 %)
Protected against at least one serotype	403 (95.0 %)	400 (93.7 %)
Protected against all serotypes	242 (57.1 %)	273 (63.9 %)

Evaluation of the above data revealed that the rate of students protected against all three serotypes increased from 57.1 % (2004–2006) to 63.9 % (2008–2010). A detailed view of the various combinations of immunity gaps against the three virus types is given in Table 1.

## Discussion

According to the strategic plan of the Global Polio Eradication Initiative (GPEI), transmission of WPV shall be stopped by the end of 2012 [10]. A high vaccination coverage is a crucial prerequisite for this goal. With an estimated coverage of 96 %, the European region holds one of the highest polio vaccination rates worldwide. The results of our seroprevalence study confirm this estimation, as 94 % of our students were antibody positive for at least one of the three poliovirus types (Table 1). One must assume that virtually all of the seropositivities were vaccine-derived rather than acquired by contact with WPV.

Although there had been no outbreak of poliomyelitis in Germany for more than a decade, the results of our study give cause for concern. Low or even unmeasurable antibody levels in vaccinated persons may well suffice to protect against disease, but nonetheless, infection with WPV or VDPV and excretion of infectious virus is possible [11]. It is, therefore, highly advisable to strictly follow the recommendation of the Standing Committee on Vaccination in Germany (STIKO) in administering a booster vaccination (with or without prior immune status control) to health care workers, especially to laboratory personnel handling stool and other potentially infectious specimens [12]. We are fairly certain that the poliomyelitis immunity status in medical students at Bonn University or at Frankfurt (Main) University [7] is at best representative for the general population in Germany, since future physicians are rarely belonging to vaccination-opposing groups.

In conclusion, there is surely a moderate improvement with regard to the poliovirus type 3 immunity in our study cohort over half a decade, but the data strongly underline the warnings by the German health authorities concerning possible importations of polioviruses [13].
